# Data-driven nitrogen application for satinleaf: leveraging optical sensors in urban landscape management

**DOI:** 10.3389/fpls.2025.1522662

**Published:** 2025-02-06

**Authors:** Bárbara Nogueira Souza Costa, Amir Ali Khoddamzadeh

**Affiliations:** Department of Earth and Environment, Institute of Environment, Florida International University, Miami, FL, United States

**Keywords:** *Chrysophyllum oliviforme*, SPAD, AtLEAF, NDVI, runoff pollution, nitrogen fertilization

## Abstract

The use of sensor technology is essential in managing fertilization, especially in urban landscape where excessive fertilization is a common issue that can lead to environmental damage and increased costs. This study focused on optimizing nitrogen fertilizer application for Satinleaf (*Chrysophyllum oliviforme*), a native Florida plant commonly used in South Florida landscaping. Fertilizer with an 8N-3P-9K formulation was applied in six different treatments: 15 g (control), 15 g (15 g twice; T1), 15 g (15 g once; T2), 30 g (15 g twice; T3), 30 g (15 g once; T4), and 45 g (15 g twice; T5). Evaluations of plant growth and nutrient status were conducted at several intervals: baseline (0), and 30, 60, 90, 120, 150, and 180 days post-fertilizer application. Three types of optical sensors-GreenSeeker™, SPAD meter, and atLEAF chlorophyll sensor - were used to monitor chlorophyll levels as an indicator of nitrogen content. The study found that the 30 g (15 g twice; T3) treatment was most effective in promoting plant growth and increasing nitrogen content in leaves and soil, while the 45 g (15 g twice; T5) treatment resulted in higher nutrient runoff, indicating potential environmental risks. These findings emphasize the value of using optical sensors for precise nitrogen management in plant nurseries to enhance growth, lower costs, and minimize environmental impact.

## 1 Introduction

Satinleaf (*Chrysophyllum oliviforme*), a medium-sized tree in the Sapotaceae family, can reach heights of up to 45 feet and has a spread of approximately 25 feet. It is renowned for its unique and attractive foliage. Native to Florida, satinleaf is a prized choice in South Florida landscaping, often featured as a standout lawn specimen or integrated into shrub borders and naturalized settings (Gilman et al., 2019). Its popularity in urban landscaping is due to its aesthetic appeal and versatility in various environments (Meerow et al., 2020). In urban landscapes, satinleaf plays a crucial role by providing shade, enhancing air quality, and adding significant visual appeal to cityscapes.

Achieving optimal nitrogen (N) fertilization involves balancing nutrient supply with plant demand, a task complicated by the difficulty of accurately predicting both factors (Colaço and Bramley, 2018; Khoddamzadeh and Dunn, 2016; Fallahi et al., 2000). Satinleaf trees have moderate to high nitrogen needs and perform best in soils enriched with organic matter (Koeser et al., 2017). Therefore, tools like optical sensors are essential for managing nitrogen levels effectively.

Typically, only a small fraction of applied nitrogen is absorbed by crops, with the excess prone to environmental loss, leading to various ecological issues. Unused nitrogen can leach below the root zone or be lost through runoff (Randall and Goss, 2008; Cameron et al., 2013; Freidenreich et al., 2019), resulting in nitrate (NO_3_^−^) accumulation in natural water bodies (Pulido-Bosch et al., 2000; Ju et al., 2006). Elevated nitrate levels in water bodies are linked to human health risks (Addiscott, 1996) and eutrophication (Cameron et al., 2013). Furthermore, excess nitrogen contributes to environmental degradation through nitrous oxide (N_2_O) emissions, which exacerbate global warming, and ammonia (NH_3_) volatilization, which enriches natural ecosystems with nitrogen (Hartz, 2006; Meisinger et al., 2015; Galloway et al., 2008; Tilman et al., 2001).

Environmental protection and water pollution, especially in South Florida with its high precipitation rates and the prevalence of harmful algal blooms, have become critical issues. The restoration of the Everglades, a unique and vital ecosystem, has taken on added importance. This region faces significant challenges due to nutrient runoff, which contributes to ecological degradation and water quality concerns. Sustainable nitrogen management practices are therefore essential to safeguard the Everglades and surrounding water bodies.

Optical sensor technology has emerged as a key tool for optimizing nitrogen fertilization and mitigating its environmental impact. These sensors enable non-destructive, efficient assessments of crop nitrogen status, supporting informed fertilizer management decisions. Among the most widely used tools are the GreenSeeker™ handheld sensor (Trimble Navigation Ltd., CA), the SPAD meter (SPAD-502, Konica Minolta, Japan), and the atLEAF chlorophyll sensor (FT Green LLC, DE) (Basyouni et al., 2015; Khoddamzadeh et al., 2023).

The GreenSeeker™ sensor calculates the normalized difference vegetation index (NDVI) using active red and near-infrared light, providing insights into plant health and nitrogen levels. The SPAD meter evaluates chlorophyll content by analyzing light transmittance through leaves, offering a reliable indicator of nitrogen concentration. Similarly, the atLEAF chlorophyll sensor provides chlorophyll readings with the added benefit of digital integration for enhanced data analysis. These tools are not only adaptable across various growth stages but also require minimal labor, making them practical for large-scale implementation (Fox and Walthall, 2015; Tremblay et al., 2012).

This study aimed to determine the optimal nitrogen fertilizer dose for satinleaf by monitoring chlorophyll content using optical sensor technology. Additionally, it sought to evaluate the correlation between sensor readings and nitrogen content in satinleaf plants, identifying the fertilizer rate that minimizes nutrient runoff and supports sustainable agriculture in South Florida.

## 2 Materials and methods

### 2.1 Plant material and growing conditions

Satinleaf *(Chrysophyllum oliviforme)* plants were sourced from Santa Barbara Nursery in Miami, Florida. The potted plants were repotted and kept under shade house conditions at the Organic Garden of Florida International University (FIU). A slow-release fertilizer with an NPK 8-3-9 formulation (Harrell’s^®^) was first applied in October, followed by various supplementary treatments.

### 2.2 Fertilizer treatments

Six different fertilizer treatments were employed: control (15-0-0), Treatment 1 (15-15-15), Treatment 2 (15-15-0), Treatment 3 (30-15-15), Treatment 4 (30-15-0), and Treatment 5 (45-15-15). Treatments were designed to evaluate the effects of varying nitrogen application rates on both plant growth and environmental impact (**Table 1**).

**Table 1:** Summary of fertilizer treatments, combinations and supplementary applications (SA) with corresponding dosages.

Treatments	Dosages	SA	Number and month of Application (SFT)
Control	15g	––––	––––
T1	15g	15g	2 - November and March
T2	15g	15g	1 - November
T3	30g	15g	2 - November and March
T4	30g	15g	1 - November
T5	45g	15g	2 - November and March

Assessments were conducted at seven timepoints: the baseline (day 0), and monthly intervals thereafter, up to 180 days post-fertilizer application (days 30, 60, 90, 120, 150, and 180). Parameters evaluated included plant growth, chlorophyll content (non-destructive), nitrogen and carbon concentration in leaf and substrate samples, as well as electrical conductivity and total nitrogen in leachate samples.

### 2.3 Growth assessment

Growth was monitored monthly by measuring the leaf count and the height of the plants. Five plants were selected from each treatment group for these measurements. Plant height was calculated by averaging the measurements from one larger and one smaller branch per plant, which were marked at the beginning of the experiment for consistency.

### 2.4 Chlorophyll content measurement

Chlorophyll content was determined using three types of devices: GreenSeeker™ NDVI sensor (Trimble Agriculture, Sunnyvale, CA, USA), SPAD-502 chlorophyll meter (Konica Minolta, Japan), and atLEAF chlorophyll meter (Wilmington, DE, USA), and. Measurements for SPAD and atLEAF were taken from four mature leaves located in the middle section of each plant. The GreenSeeker™ sensor was placed 45 cm above the plant canopy to ensure uniform readings. Five plants were used from each treatment for these measurements.

### 2.5 Analysis of nitrogen/carbon content in soil and leaves

Leaf samples were collected every month from each treatment, while soil samples were obtained at the beginning and end of the experiment. Both leaf and soil samples were dried at 70°C for 48 hours, ground to a fine consistency, and then analyzed for total nitrogen and carbon content using standard protocols at FIU’s Center for Aquatic Chemistry and Ecotoxicology (CAChE) Nutrient Analysis Core Facility (SOP-012). The analysis was conducted following the Carlo Erba NA1500 Series 1 Operating Manual, the Standard Operating Procedure for Instrumental Analysis for Total Organic Carbon and Total Nitrogen in Sediments (USGS Reston, Virginia Environmental Organic Geochemistry Laboratory, 13pp), and EPA Method 440.0 (Determination of Carbon and Nitrogen in Sediments and Particulates of Estuarine/Coastal Waters Using Elemental Analysis, U.S. Environmental Protection Agency, Washington, DC, EPA/600/R-15/009, 1997). Five plants were used to collect these samples.

### 2.6 Runoff collection and nutrient analysis

Runoff was collected using individual containers placed under each 3 gallons pot (Dimensions: 11” wide x 9.5” tall) during irrigation. Five plants from each treatment were used to collected leachate samples from each treatment, the plants were watered to saturation, and a further 350 ml of water was added to each pot to generate leachate. The total leachate collected was used to measure electric conductivity and salt content in situ. A 50 ml aliquot of the leachate was immediately refrigerated at 4°C and later analyzed for total nitrogen at the CAChE Nutrient Analysis Core Facility.

### 2.7 Statistical analysis

The experiment was conducted as a completely randomized design (CRD) consist of six treatments, each replicated five times, for a total of 30 plants (one per pot). Data were analyzed using ANOVA, and differences between treatment means were evaluated using Tukey’s test at a 5% significance level through the SISVAR statistical program (Ferreira, 2011). Correlation analyses were conducted between sensor readings, leaf number, and nitrogen and carbon content in the leaves using GraphPad Prism software (v. 9.4.1, GraphPad, San Diego, CA, USA).

## 3 Results

### 3.1 Growth and chlorophyll content analysis

The data analysis revealed no significant (p > 0.05) association between fertilization rates and evaluation periods regarding the leaf number and Normalized Difference Vegetation Index (NDVI) values. Therefore, these factors were examined independently. The various fertilizer treatments did not significantly impact the number of leaves or NDVI values (p > 0.05). However, notable differences were observed in chlorophyll content as measured by the SPAD and atLEAF sensors (p ≤ 0.05). The T5, T3, and control showed increased SPAD values (77.08, 76.91, and 77.39, respectively), compared to the T1, which had lower values (73.80). Similarly, the atLEAF readings were higher for the T2 (75.91) compared to the T1 (72.67) and T3 (73.73). The T5 also showed elevated atLEAF readings (75.33) in comparison to the T1 (72.67) (**Table 2**).

**Table 2:** Number of leaves, chlorophyll Levels (SPAD and atLEAF Measurements), and NDVI values of satinleaf plants under different fertilizer treatments (FT).

Treatments	Number of Leaves (unit)	SPAD	atLEAF	NDVI
Control	96.26 a	77.39 a	74.71 abc	0.83 a
T1	112.77 a	73.80 b	72.67 c	0.83 a
T2	94.51 a	75.26 ab	75.91 a	0.82 a
T3	118.31 a	76.91 a	73.73 bc	0.84 a
T4	109.14 a	76.17 ab	74.66 abc	0.82 a
T5	90.37 a	77.08 a	75.33 ab	0.83 a

Means followed by the same letter lower case in the columns (Treatments) and upper case in the rows (DPFA) are not significantly different by Tukey’s test (p ≤ 0.05). Control (15-0-0), Treatment 1 (15-15-15), Treatment 2 (15-15-0), Treatment 3 (30-15-15), Treatment 4 (30-15-0), and Treatment 5 (45-15-15).

### 3.2 Effect of days post-fertilizer application on chlorophyll content

The number of leaves remained unaffected by days post-fertilizer application (DPFA) (p > 0.05). However, the chlorophyll content, as measured by SPAD and atLEAF, and NDVI values varied significantly over time (p ≤ 0.05). The highest SPAD values were recorded at 90 (78.89), 150 (77.04), and 180 days post-fertilizer application (DPFA) (78.94) compared to 0 DPFA (72.77). The highest atLEAF readings occurred at 60 (76.36) and 180 DPFA (76.27), surpassing readings at 30 (73.38), 90 (73.00), and 120 DPFA (72.88). The lowest NDVI value was observed at 180 DPFA (0.73), which was significantly lower than the values recorded at 0 (0.84), 30 (0.84), 60 (0.86), 90 (0.85), 120 (0.86), and 150 DPFA (0.82) (**Table 3**).

**Table 3:** Changes in number of leaves (NL), chlorophyll content (SPAD and atLEAF), and NDVI across days post-fertilizer application (DPFA) in satinleaf plants.

DPFA	NL (unit)	SPAD	atLEAF	NDVI
0	90.40 a	72.77 b	74.04 ab	0.84 a
30	91.60 a	74.95 ab	73.38 b	0.84 a
60	180.80 a	75.17 ab	76.36 a	0.86 a
90	96.47 a	78.89 a	73.00 b	0.85 a
120	105.73 a	74.95 ab	72.88 b	0.86 a
150	104.97 a	77.04 a	75.58 ab	0.82 a
180	104.97 a	78.94 a	76.27 a	0.73 b

Means followed by the same letter within columns are not significantly different by Tukey’s test (p ≤ 0.05).

### 3.3 Impact of fertilizer treatments on plant height and nutrient content

**Figure 1:**
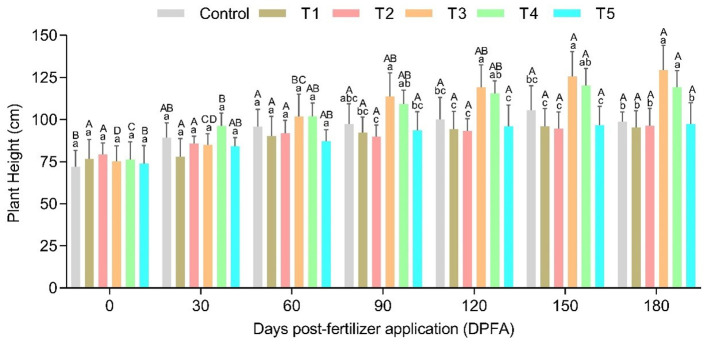
Plant height of satinleaf plants grown at Different Fertilization Levels and Days Post-Fertilizer Application (DPFA). Control (15-0-0), Treatment 1 (15-15-15), Treatment 2 (15-15-0), Treatment 3 (30-15-15), Treatment 4 (30-15-0), and Treatment 5 (45-15-15). Means followed by the same letter lower case (Treatments) and upper case (DPFA) are not significantly different by Tukey’s test (p ≤ 0.05).

A significant (p ≤ 0.05) association was found between fertilization rate and DPFA for plant height in Satinleaf plants. The highest increases in plant height were observed in the T3 and T4, reaching 129.40 cm and 119.40 cm, respectively, at 180 DPFA (**Figure 1**). Furthermore, there was significant (p ≤ 0.05) association between fertilization rate and DPFA for total nitrogen (TN) and total carbon (TC) in leaf samples. The T4 achieved the highest total nitrogen concentration (2.95%) at 60 DPFA, while the highest total carbon content (49.33%) was recorded in the T1 at 150 DPFA (**Table 4**).

**Table 4:** Total nitrogen, and total carbon in satinleaf plants across various fertilizer treatments and days post-fertilizer application (DPFA).

Treatments	DPFA						
	0	30	60	90	120	150	180
	Total Nitrogen (% dry leaf mass)						
Control	1.46 aA	1.46 fA	0.91 fF	1.16 fD	1.18 fC	1.14 fE	1.24 fB
T1	1.46 aG	1.82 cA	1.79 dB	1.68 eE	1.62 dF	1.73 eD	1.78 dC
T2	1.46 aF	1.80 dC	1.85 cB	1.96 cA	1.79 cD	1.79 dD	1.58 eE
T3	1.46 aG	1.62 eE	1.67 eD	1.72 dC	1.61 eF	1.95 cA	1.80 cB
T4	1.46 aF	2.72 aC	2.95 aA	2.75 aB	2.20 bE	2.72 aC	2.24 aD
T5	1.46 aF	2.00 bE	2.27 bB	2.03 bD	2.30 aA	2.30 bA	2.06 bC

	0	30	60	90	120	150	180
	Total Carbon (% dry leaf mass)						
Control	46.97 aC	46.97 cC	39.37 fF	47.41 cA	47.36 aB	46.43 fD	46.27 bE
T1	46.97 aD	46.89 dF	46.94 aE	47.53 bB	47.23 bC	49.33 aA	46.02 cG
T2	46.97 aB	48.18 aA	43.77 cG	46.93 dC	46.71 dE	46.72 eD	45.36 dF
T3	46.97 aE	47.35 bC	42.54 eG	48.74 aA	47.08 cD	48.52 bB	44.62 eF
T4	46.97 aC	46.05 fF	44.97 bG	46.42 eE	46.63 fD	48.43 cA	47.53 aB
T5	46.97 aB	46.78 eC	43.26 dG	45.44 fE	46.67 eD	47.98 dA	43.61 fF

Means followed by the same letter lower case in the columns (Treatments) and upper case in the rows (DPFA) are not significantly different by Tukey’s test (p ≤ 0.05). Control (15-0-0), Treatment 1 (15-15-15), Treatment 2 (15-15-0), Treatment 3 (30-15-15), Treatment 4 (30-15-0), and Treatment 5 (45-15-15).

### 3.4 Soil nutrient content and runoff analysis

Significant (p ≤ 0.05) association were also found between fertilizer rate and DPFA for nitrogen and carbon levels in soil samples. For instance, the T3 resulted in the highest total nitrogen (1.43%) and carbon content (33.91%) in the soil at 180 DPFA (**Table 5**). Runoff analysis indicated a significant (p ≤ 0.05) correlation between fertilization rate and DPFA for salt content, electrical conductivity (EC), and total nitrogen (TN) in leachate samples. The T5 showed the highest levels of salt (2952 ppm), EC (5502 µs), and TN (192 ppm) at 30 DPFA, indicating substantial nutrient loss through runoff (**Table 6**).

**Table 5:** Total nitrogen, and total carbon in satinleaf cultivation at different fertilization levels measured at baseline and 180 days post-fertilizer application (DPFA).

Treatments	DPFA	
	0	180
	Total Nitrogen (% dry soil mass)	
Control	1.07 aB	1.19 dA
T1	1.07 aB	1.20 cA
T2	1.07 aA	1.05 f B
T3	1.07 aB	1.43 aA
T4	1.07 aB	1.18 eA
T5	1.07 aB	1.33 bA

	0	180
	Total Carbon (% dry soil mass)	
Control	28.60 aA	27.40 eB
T1	28.60 aA	26.62 fB
T2	28.60 aB	30.11 bA
T3	28.60 aB	33.91 aA
T4	28.60 aA	28.07 dB
T5	28.60 aB	29.47 cA

Means followed by the same letter lower case in the columns (Treatments) and upper case in the rows (DPFA) are not significantly different by Tukey’s test (p ≤ 0.05). 15g (control), 15g (15g applied twice in November and March; T1), 15g (15g November; T2), 30g (15g applied twice in November and March; T3), 30g (15g November; T4) and 45g (15g applied twice in November and March; T5).

**Table 6:** Leachate analysis of salt content, electrical conductivity, and total nitrogen in satinleaf plants under days post-fertilizer application (DPFA).

Treatments	DPFA						
	0	30	60	90	120	150	180
	Salt (ppm)						
Control	289.00 aB	1232.60 dA	541.60 dB	669.20 bAB	395.40 aB	463.20 bB	453.20 bcB
T1	289.00 aB	1974.00 bcA	1108.80 bcdB	1165.80 abB	636.00 AbcBC	1766.00 Aa	984.40 abB
T2	289.00 aB	1596.00 cdA	757.80 cdB	802.40 Bb	529.00 aB	441.40 bB	408.20 cB
T3	289.00 aC	2192.00 bA	1140.80 bcB	736.20 bBC	469.60 aC	1296.00 aB	765.40 abcBC
T4	289.00 aD	2142.00 bcA	1410.60 abB	1149.60 abBC	594.00 aCD	544.60 bD	434.80 bcD
T5	289.00 aE	2952.00 aA	1743.60 aB	1396.20 aBC	621.00 aDE	1664.00 aB	1046.20 aCD

	0	30	60	90	120	150	180
	Electrical Conductivity (µs/cm)						
Control	603.80 aA	2403.20 dB	1091.00 dB	1357.40 bAB	816.60 aB	949.60 bB	931.00 bB
T1	603.80 aC	3762.00 bcA	2214.20 bcB	2265.00 abB	1294.00 aBC	3364.00 aA	2059.40 aB
T2	603.80 aB	3110.00 cdA	1514.80 cdB	1607.60 bB	1063.40 aB	710.40 bB	842.60 bB
T3	603.80 aC	4172.00 bA	2199.80 bBc	1467.00 bBC	963.40 aC	2518.00 aB	1544.40 abBC
T4	603.80 aD	3988.00 bcA	2725.00 abB	2245.00 abBC	1210.40 aCD	1118.40 bD	899.00 bD
T5	603.80 aE	5502.00 aA	3354.40 aB	2734.00 aBC	1253.20 aDE	3170.00 aB	2053.80 aCD

	0	30	60	90	120	150	180
	Total Nitrogen (ppm)						
Control	3.03 aG	45.83 fA	8.73 fC	6.17 dE	5.73 fF	8.20 eD	13.55 eB
T1	3.03 aG	91.50 eA	40.50 cC	10.50 aE	6.23 eF	12.63 dD	44.90 cB
T2	3.03 aG	99.50 dA	12.75 eD	10.20 bE	47.67 aB	6.25 fF	20.66 dC
T3	3.03 aG	101.00 cA	38.50 dD	9.13 cF	9.45 dE	78.83 aB	45.41 bC
T4	3.03 aG	134.00 bA	77.75 bB	3.77 fF	13.00 cD	14.62 bC	8.30 fE
T5	3.03 aG	192.00 aA	108.25 aB	3.97 eF	16.77 bD	12.67 cE	58.40 aC

Means followed by the same letter lower case in the columns (Treatments) and upper case in the rows (DPFA) are not significantly different by Tukey’s test (p ≤ 0.05). Control (15-0-0), Treatment 1 (15-15-15), Treatment 2 (15-15-0), Treatment 3 (30-15-15), Treatment 4 (30-15-0), and Treatment 5 (45-15-15).

### 3.5 Correlation analysis

Correlation analysis demonstrated strong negative correlations between SPAD readings and total carbon at 60 DPFA (-0.858) and between total nitrogen and total carbon at both 120 DPFA (-0.905) and 180 DPFA (-0.819). Additionally, a strong negative correlation was observed between SPAD and NDVI at 120 DPFA (-0.986). These correlations highlight the complex interplay between different parameters under varying fertilizer treatments (**Table 7**).

**Table 7:** Pearson’s correlation coefficients (*r*) between sensor parameters, total nitrogen (TN), and total carbon (TC) in satinleaf plants measured across days post-fertilizer application (DPFA).

DPFA	Variables	Correlation Value	Significance
60	SPAD vs. TC (%)	-0.858	p≤ 0.05
120	SPAD vs. NDVI	-0.986	p ≤ 0.001
120	TN (%) vs. TC (%)	-0.905	p≤ 0.01
180	TN (%) vs. TC (%)	-0.819	p≤ 0.05

## 4 Discussion

Nitrogen is a vital nutrient that significantly influences plant growth and development, primarily as a core component of chlorophyll in leaves. Chlorophyll levels directly affect leaf area, biomass, plant height, and water usage. Inadequate nitrogen can cause deficiency symptoms that negatively impact plant health, productivity, and commercial value. On the other hand, excessive nitrogen application can lead to toxicity, resulting in stunted growth and poor plant quality. Over-application also raises production costs and poses environmental risks due to nutrient leaching and runoff, leading to contamination of water bodies and ecosystems (Khoddamzadeh and Dunn, 2016; Basyouni and Dunn, 2013). This study utilized optical sensors as a non-destructive method to estimate chlorophyll content and assess nitrogen status, aiming to identify the optimal fertilizer dosage for Satinleaf that balances growth and environmental sustainability.

The results indicated that the T3 effectively promoted Satinleaf growth, as evidenced by an increase in plant height and higher chlorophyll content measured by SPAD. This treatment also enhanced total nitrogen levels in both the leaves and soil substrate. These findings are consistent with those reported by Costa et al. (2023) for Cocoplum plants, where treatment 3 (30-15-15) treatment was also found to be the optimal nitrogen dose for promoting growth without excessive nutrient loss. These outcomes suggest that moderate nitrogen doses can provide sufficient nutrients for optimal plant growth while minimizing nutrient loss through runoff.

The results also show that the Control treatment provided higher values for SPAD, compared with the with higher doses. This may have happened due to high doses of nitrogen can generate severe vegetative growth, which can lead to the dilution effect where the concentration of chlorophyll per leaf unit decreases due to the increase in leaf area. This can also explain the why T4 has higher N concentrations in the leaf dry mass compared to T5.

The T4 achieved the highest total nitrogen concentration at 60 DPFA, while the highest total carbon content was recorded in the T1 at 150 DPFA. Furthermore, T1 also provided lower chlorophyll values (SPAD and atLEAF) compared to the other treatments, which shows that this plant was efficient in absorbing carbon, but was not efficient in converting it into chlorophyll through photosynthesis.

As observed in this study, chlorophyll content measured by SPAD and atLEAF sensors increased over time following fertilizer application, while NDVI values showed a decline towards the end of the experiment. The highest fertilizer dose T5, resulted in elevated salt concentration, electrical conductivity (EC), and total nitrogen levels in the runoff samples. This indicates substantial nitrogen loss due to runoff, which can harm the environment. A more sustainable approach to nitrogen management involves using moderate doses, such as the T3, which supports effective plant growth while reducing the risk of environmental contamination. Similar patterns were observed in studies on other native and non-native plants in South Florida, including Cocoplum (Costa et al., 2023) and Cacao (Khoddamzadeh and Souza Costa, 2023).

In general, all plants were watered manually, which may have resulted in different amount for each plant. However, for the nutrient runoff analysis, a methodology was followed in which the size of the pots was considered and the same amount was added to all the plants, in addition the saturation point was the same for all treatments and the water was observed the flowing through the pots. Therefore, the large amount of nutrient runoff may have been primarily due to the amount of fertilizers applied, thus showing the difference between the high fertilizer treatments and the control.

Correlation analysis revealed significant negative correlations between SPAD readings and total carbon at 60 days post-fertilizer application (DPFA) and between total nitrogen and total carbon at 120 and 180 DPFA. A strong negative correlation was also found between SPAD and NDVI. These negative correlations suggest that as one parameter increases, the other decreases, reflecting the complex correlation between these variables. Khoddamzadeh and Souza Costa (2023) also noted similar negative correlations between total nitrogen and total carbon in Cacao plants grown under different fertilization rates, further emphasizing the need to balance these nutrients for optimal plant health and growth.

## 5 Conclusions

This study demonstrates that Satinleaf (Chrysophyllum oliviforme) plants can thrive with lower nitrogen fertilizer doses, which also help mitigate nutrient runoff pollution. The treatment with 30 g (15 g applied twice in November and March; T3) proved to be the most effective, enhancing plant growth and nitrogen levels in both soil and plant tissues. In contrast, the 45 g (15 g applied twice; T5) treatment resulted in substantial nutrient loss through runoff, making it less ideal for sustainable urban landscape management.

The findings underscore the importance of integrating sensor technologies, such as GreenSeeker™, SPAD meter, and atLEAF chlorophyll sensors, into fertilization management strategies. These tools enable precise monitoring of nitrogen levels, optimizing fertilization to reduce environmental impacts, minimize costs, and prevent over-fertilization.

Future research should focus on improving the sensitivity and accessibility of sensor technology to better serve landscape professionals. Additionally, further investigations into how sensor-based fertilization management can be adapted to diverse urban environments and plant species will enhance its practical application. These advancements can contribute significantly to more sustainable urban landscape practices and environmental resilience.

## Data Availability

The raw data supporting the conclusions of this article will be made available by the authors, without undue reservation.
